# The Anti-Atherosclerosis Effect of Anakinra, a Recombinant Human Interleukin-1 Receptor Antagonist, in Apolipoprotein E Knockout Mice

**DOI:** 10.3390/ijms23094906

**Published:** 2022-04-28

**Authors:** Eu Jeong Ku, Bo-Rahm Kim, Jee-In Lee, Yun Kyung Lee, Tae Jung Oh, Hak C. Jang, Sung Hee Choi

**Affiliations:** 1 Department of Internal Medicine, Chungbuk National University Hospital, Cheongju 28644, Korea; eujeong.ku@gmail.com; 2 Department of Internal Medicine, Chungbuk National University College of Medicine, Cheongju 28644, Korea; 3 Department of Internal Medicine, Seoul National University Bundang Hospital, Seongnam 13620, Korea; cjlovem@naver.com (B.-R.K.); dlwldls715@naver.com (J.-I.L.); leeykyung@gmail.com (Y.K.L.); ohtjmd@gmail.com (T.J.O.); janghak@snu.ac.kr (H.C.J.); 4 Department of Internal Medicine, Seoul National University College of Medicine, Seoul 03080, Korea

**Keywords:** atherosclerosis, IL-1 receptor blocker, anakinra, smooth muscle cell migration, anti-inflammation

## Abstract

Interleukin (IL)-1β plays an important role in atherosclerosis pathogenesis. We aimed to investigate the effect of anakinra, a recombinant human IL-1 receptor antagonist, on the progression of atherosclerosis in apolipoprotein E knockout (ApoE^–/–^) mice. ApoE^–/–^ mice (8-week male) were treated with saline (control), anakinra 10, 25, and 50 mg/kg, respectively (*n* = 10 in each group). Mice were fed a standard chow (4 weeks) followed by an atherogenic diet (35kcal% fat, 1.25% cholesterol, 12 weeks). Atheromatous plaques in ApoE^–/–^ mice and the expression of inflammatory genes and signaling pathways in human umbilical vein endothelial cells (HUVECs), rat aortic smooth muscle cells (RAOSMCs), and 3T3-L1 adipocytes were assessed. Anakinra reduced the plaque size of the aortic arch (30.6% and 25.2% at the 25 mg/kg and 50 mg/kg doses, both *p <* 0.05) and serum triglyceride in ApoE^–/–^ mice and suppressed inflammatory genes (IL-1β and IL-6) expressions in HUVECs and RAOSMCs (all *p <* 0.05). In RAOSMCs, anakinra reduced metalloproteinase-9 expression in a dose-dependent manner and inhibited cell migration. Anakinra-treated mice exhibited trends of lower CD68+ macrophage infiltration in visceral fat and monocyte chemoattractant protein-1 expression was reduced in 3T3-L1 adipocytes. Anakinra could be a useful component for complementary treatment with a standard regimen to reduce the residual cardiovascular risk.

## 1. Introduction

Atherosclerotic cardiovascular diseases (CVDs) are the major cause of mortality worldwide [1]. There is widespread evidence to support the use of high-intensity statin treatment to reduce low-density lipoprotein cholesterol (LDL-C) effectively as the first-line treatment for atherosclerosis. However, the residual risk for developing cardiovascular events after statin treatment remains up to 50–60%. The accumulating body of evidence in humans and animals suggests that chronic inflammation plays a critical role in the process of atherosclerosis [2]. Beyond the roles of lipid-lowering therapy with statins, ezetimibe, and pro-protein convertase subtilisin/kexin type 9 inhibitor, the effective inhibition of chronic inflammation may be an important component of anti-atherosclerosis treatment.

Interleukin (IL)-1β is an important mediator of inflammatory responses, driving the expression of mediators such as cyclooxygenase-2, IL-1, IL-6, IL-12, intercellular adhesion molecule-1 (ICAM-1), vascular cell adhesion molecule-1, and the tumor necrosis factor-α (TNF-α) signaling pathway, which all contribute to the development of vascular remodeling and atherosclerosis [3,4,5,6]. The inflammasome is an intracellular multiprotein complex that activates a pro-inflammatory cascade in response to signals from microbe-derived pathogen-associated molecular patterns and host cell-generated danger-associated molecular patterns [7]. Notably, intracellular protein NACHT, LRR, and PYD domain-containing protein 3 (NLRP3) form the NLRP3 inflammasome, which mediates the effects of IL-1β related to atherosclerosis. This process causes an induction of the expression of various inflammatory cytokines and chemokines, an increased expression of leukocyte adhesion molecules in endothelial cells, the activation of cell proliferation, remodeling and migration of molecules in smooth muscle cells, and the alteration of monocytes/macrophages involved in innate immunity [8].

Based on these findings, several trials have been conducted with the aim of applying anti-inflammatory therapy targeting the ligands or receptors for the IL-1 family to treat CVD [9,10,11]. Anti-IL-1 agents have shown particularly promising results in basic and translational studies, and further, have been reported to have effects on CVD including acute myocardial infarction (MI), heart failure, and pericarditis [12,13,14].

Anakinra, a recombinant human IL-1 receptor antagonist that blocks the biological cascades of IL-1, has applications in the reduction of systemic inflammatory responses. In 2001, it was approved by the US Food and Drug Administration for the treatment of rheumatoid arthritis, and has shown significant therapeutic effects on a range of systemic autoimmune diseases such as cryopyrin-associated periodic syndromes and juvenile and adult onset Still’s disease [15]. Several small clinical trials using anakinra as a putative treatment strategy for CVD rather than for systemic autoimmune diseases have been reported [16,17,18]. In these previous studies, anakinra produced relatively short-term changes in inflammatory biomarkers, or an improvement in cardiac function. However, the use of anakinra to treat atherosclerosis requires further validation and requires an investigation of the mechanistic role of anakinra in the progression of atherosclerotic CVD.

This study aimed to investigate the anti-atherosclerosis effects of chronic (16 weeks) treatment with anakinra to block IL-1 activity in apolipoprotein E knockout (ApoE^–/–^) mice fed an atherogenic diet, and to explore its possible mechanisms of action.

## 2. Results

### 2.1. Focus on Atherosclerosis

#### 2.1.1. Anakinra Reduces the Atherosclerotic Plaque Area in ApoE^–/–^ Mice

**Figure 1:**
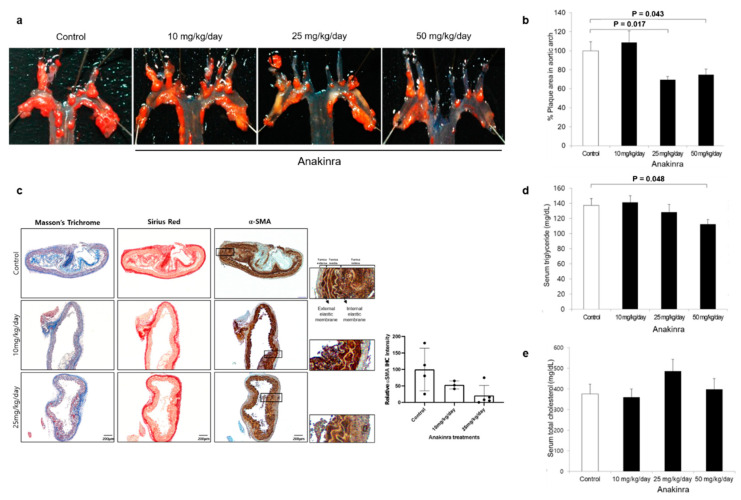
The effect of anakinra on atherosclerosis. (**a**) Area of atheromatous plaque in the aortic arch in ApoE^–/–^ mice was measured using the *en face* method and expressed as a percentage of oil-red O-positive pixels compared with the control group. (**b**) Plaque area was significantly reduced by 30.6% and 25.2% compared with the control group by treatment with 25 mg/kg and 50 mg/kg anakinra, respectively. (**c**) Representative images of plaques stained with Masson’s trichrome, Sirus red, and α-SMA (scale bar represents 200 μm). The area of α-SMA staining was further magnified corresponding to their representative images (scale bar represents 50 μm). (**d**) In ApoE^–/–^ mice, serum triglyceride levels decreased following treatment with 50 mg/kg anakinra. (**e**) In ApoE^–/–^ mice, serum total cholesterol did not differ significantly between groups. Each group was compared with the control group (anakinra 0 ng/mL) using the analysis of variance (ANOVA) with Tukey’s post hoc analysis for multiple group comparison.

Images of aortic arches with atheromatous plaque from representative individuals of each group are shown in Figure 1. Atheromatous plaque accumulation in the aortic arch of atherogenic-dieted ApoE^–/–^ mice was reduced by 30.6% and 25.2% at the 25 and 50 mg/kg doses of anakinra, respectively (both *p* < 0.05, compared with vehicle-treated ApoE^–/–^ mice). An histopathological analysis using Masson’s trichrome, Sirus red and alpha-smooth muscle actin (α-SMA) showed a significant decrease in the volume of plaques, fibrous cap, the collagen content and depositions of smooth muscle cell of the anakinra treated groups compared to the control group. Serum triglycerides (TG) were significantly decreased in mice treated with 50 mg/kg anakinra (*p* < 0.05), whereas there was no change in serum total cholesterol.

#### 2.1.2. The Dose-Dependent Effect of Anakinra on the Activation of the NLRP3 Inflammasome and Upregulated Expression of Inflammatory Adhesion Molecules in Human Umbilical Vein Endothelial Cells (HUVECs)

**Figure 2:**
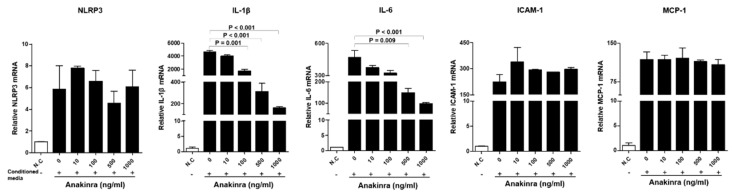
The effect of dose-dependent anakinra on the activated NLRP3 inflammasome and upregulated expression of inflammatory molecules in HUVECs after stimulation with conditioned media from differentiated LPS and TNF-α stimulated THP-1 macrophages. Gene expressions were analyzed with qRT-PCR. Each group was compared with the control group (anakinra 0 ng/mL) using an analysis of variance (ANOVA) with Tukey’s post hoc analysis for multiple group comparison.

Expression of mRNAs for NLRP3, IL-1β, IL-6, ICAM-1, and monocyte chemoattractant protein-1 (MCP-1) were significantly increased in HUVECs stimulated with the conditioned medium from differentiated lipopolysaccharide (LPS) and TNF-α-stimulated THP-1 macrophages compared with unmanipulated control HUVECs (all *p* < 0.001) (Figure 2). The expression of each mRNA in stimulated HUVECs after each dose of anakinra was compared with the positive control (without anakinra treatment). The expression of NLRP3 mRNA was not significantly changed by treatment with anakinra. The expression of IL-1β mRNA markedly decreased following the administration of anakinra doses of 100 ng/mL, 500 ng/mL, and 1000 ng/mL, respectively (all *p* < 0.01). IL-6 mRNA expression was also significantly decreased at high concentrations of anakinra (500 ng/mL and 1000 ng/mL, both *p* < 0.01). However, the expression of ICAM-1 and MCP-1 mRNAs in HUVECs was not significantly changed by anakinra. Western blot analyses showed that IL-1β, TNF-α, and ICAM-1 tended to decrease in anakinra treatment groups (Supplementary Figure S1).

#### 2.1.3. The Dose-Dependent Effect of Anakinra on the Activated Nlrp3 Inflammasome and Upregulated Expression of Angiogenesis Molecules in Rat Aortic Smooth Muscle Cells (RAOSMCs)

**Figure 3:**
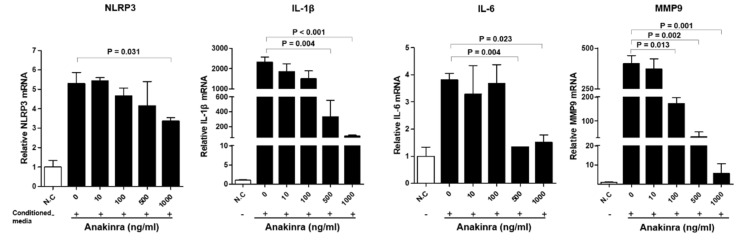
The effect of dose-dependent anakinra on the activated NLRP3 inflammasome and upregulated expression of inflammatory molecules in RAOSMCs after stimulation with conditioned media from differentiated LPS and TNF-α stimulated THP-1 macrophages. Gene expressions were analyzed with qRT-PCR. Each group was compared with the control group (anakinra 0 ng/mL) using the analysis of variance (ANOVA) with Tukey’s post hoc analysis for multiple group comparison.

Stimulation of RAOSMCs with the supernatants from LPS- and TNF-α-stimulated differentiated THP-1 macrophages induced a significantly increased expression of mRNAs for NLRP3, IL-1β, IL-6, and matrix metalloproteinase-9 (MMP-9). RAOSMCs treated with relatively high doses of anakinra showed a significant decrease in expression of NLRP3 mRNA (1000 ng/mL), IL-1β (500 ng/mL and 1000 ng/mL), and IL-6 (500 ng/mL and 1000 ng/mL) compared with no anakinra treatment (all *p* < 0.05) (Figure 3). The expression of MMP-9 was dose dependently reduced with 100 ng/mL, 500 ng/mL, and 1000 ng/mL doses of anakinra (all *p* < 0.05, compared with the no anakinra treatment group). Western blot analyses showed that NLRP3 inflammasome was significantly decreased in anakinra of 500 ng/mL and TNF-α and MMP-9 were significantly reduced in anakinra 10, 500 and 1000 ng/mL (Supplementary Figure S1).

#### 2.1.4. The Effect of Anakinra on the p38 Mitogen-Activated Protein Kinase (MAPK)/Nuclear Factor-κB (NF-κB) Pathway in RAOSMCs and HUVECs

**Figure 4:**
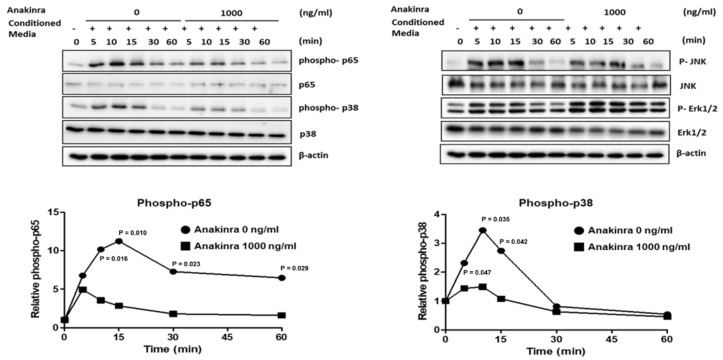
The effect of anakinra on the mitogen-activated protein kinase (MAPK)/nuclear factor-κB (NF-κB) pathway in RAOSMC. Each group was compared with the control group (anakinra 0 ng/mL) using analysis of variance (ANOVA) with Tukey’s post hoc analysis for multiple group comparison.

The results of the Western blot analysis demonstrated that treatment of RAOSMCs for 5, 10, 15, 30, or 60 min with the supernatant from LPS- and TNF-α-stimulated THP-1 macrophages induced increased expression of phospho-NF-κB p65 protein, which reached peak levels after 15 min and remained elevated until 60 min. Exposure of RAOSMCs to the supernatant from the LPS- and TNF-α-stimulated differentiated THP-1 macrophages for 60 min also induced significantly increased expression of phospho-p38 MAPK. Because NF-κB plays an important role in the regulation of the vascular inflammatory responses, we examined the effect of anakinra on the phosphorylation of NF-κB p65, an essential step in the activation of NF-κB. Activation of the p38 MAPK/NF-κB pathway in RAOSMCs was inhibited by treatment with anakinra (1000 ng/mL) (*p* < 0.05) (Figure 4). In HUVECs, the phosphorylation of p65 and extracellular-regulated kinases (ERK) 1/2 showed a tendency to decrease in the anakinra-treated group compared with the control group, but this was not significant. Although not statistically significant in Western blot analysis, the results were similar to those of gene expression trends (data not shown).

#### 2.1.5. Anakinra Inhibits Migration of RAOSMCs

**Figure 5:**
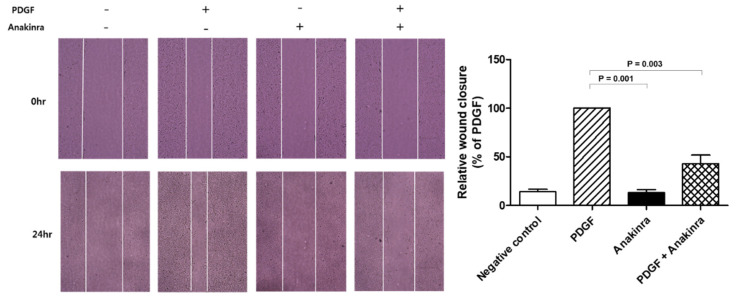
Wound healing assay in RAOSMC. The migration capacity was assessed with a wound healing assay. Anakinra reduced cell migration in RAOSMCs compared with the positive control. Results are expressed as a percentage of PDGF-stimulated migration (mean ± SD). *n* = 3 wells, 10 fields-of-view per well. Each group was compared with the positive control (PDGF) group using the analysis of variance (ANOVA) with Tukey’s post hoc analysis for multiple group comparison.

To better understand the effects of anakinra on vascular injury and repair, a migration/wound healing assay was performed using RAOSMCs stimulated by platelet-derived growth factor (PDGF). A representative field is shown in Figure 5. Migration capability measured by the wound-healing assay revealed that anakinra caused a significant reduction in RAOSMC migration of 57%.

### 2.2. Focus on Fat

#### 2.2.1. Immunofluorescent Staining of CD68 and Hematoxylin and Eosin (H&E) Staining in Visceral Adipose Tissue

**Figure 6:**
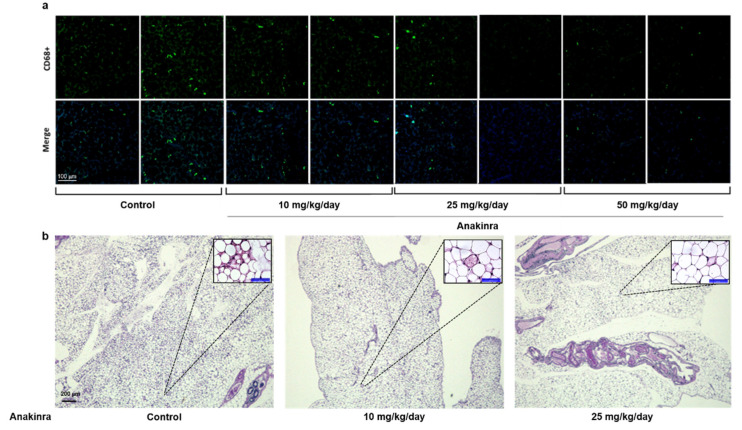
Visceral adipose tissue. (**a**) Immunofluorescence staining of CD68 (1:200, scale bars represents 100 μm) and (**b**) H&E staining in visceral adipose tissue (scale bars represents 200 μm).

Immunofluorescent staining for CD68, which is a surface marker of M1 macrophages, indicated that the number of M1 macrophages in the visceral adipose tissue of mice tended to decrease in mice treated with 25 mg/kg and 50 mg/kg anakinra compared with controls, but these differences were not significant (Figure 6a). There was a trend in the decrease in crown-like structures in adipose tissue with increasing doses of anakinra treatment, indicative of the degeneration of adipocytes surrounded by inflammatory cells (Figure 6b).

#### 2.2.2. The Dose-Dependent Effect of Anakinra on the Activated NLRP3 Inflammasome and Upregulated Expression of Inflammatory Molecules in 3T3-L1 Adipocytes

**Figure 7:**
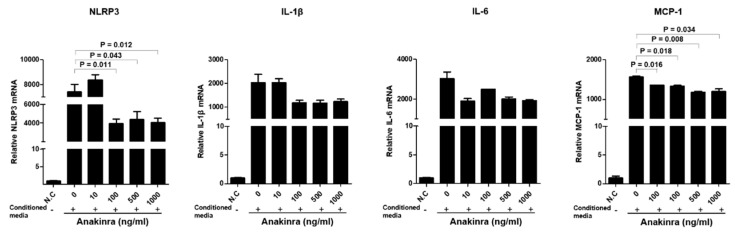
The effect of dose-dependent anakinra on the activated NLRP3 inflammasome and upregulated expression of inflammatory molecules in 3T3-L1 adipocytes after stimulation with conditioned media from differentiated LPS and TNF-α stimulated THP-1 macrophages. Gene expressions were analyzed by qRT-PCR. Each group was compared with the control group (anakinra 0 ng/mL) using the analysis of variance (ANOVA) with Tukey’s post hoc analysis for multiple group comparison.

The results of these experiments are shown in Figure 7. Compared with the negative control group, 3T3-L1 adipocytes stimulated with supernatant from LPS- and TNF-α-stimulated THP-1 macrophages showed a significant upregulation of mRNA for NLRP3, IL-1β, IL-6, and MCP-1 (all *p* < 0.001). The treatment of 3T3-L1 adipocytes with 100 ng/mL, 500 ng/mL, and 1000 ng/mL anakinra significantly reduced the levels of NLRP3 mRNA and IL-1β mRNA (all *p* < 0.05). The levels of MCP-1 mRNA were also significantly decreased for all doses of anakinra (all *p* < 0.05). IL-6 mRNAs showed a tendency to decrease at high doses of anakinra.

#### 2.2.3. Expression of Phosphorylated c-Jun N-Terminal Kinase (p-JNK), *p*-p38, and p-ERK in the Liver and Visceral Fat Tissue

The expression of p-ERK in liver tissue tended to decrease at higher doses of anakinra (25 mg/kg and 50 mg/kg/day) compared with the control group, but this was not significant. Similarly, the expression of p-ERK in visceral adipose tissue showed a nonsignificant tendency to decrease in the high-dose anakinra (50 mg/kg/day) group compared with the control group. In terms of the expression of p-JNK in fat tissue, the anakinra 25 mg/kg/day group showed an increase compared with the control group but this was not dose-dependent (Supplementary Figure S2).

## 3. Discussion

**Figure 8:**
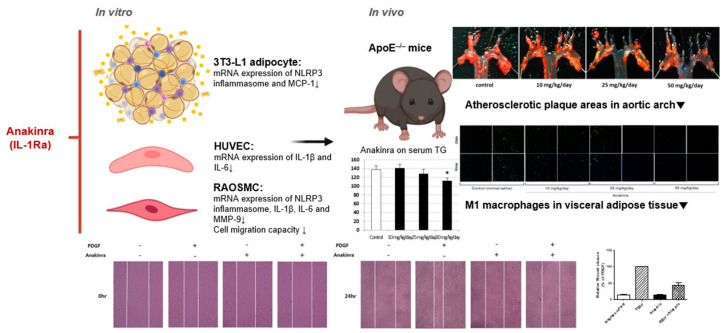
Summary of results.

This study demonstrated that IL-1 blockade with anakinra significantly reduced atherosclerotic plaque formation and progression in the aortic arch of ApoE^–/–^ mice fed an atherogenic diet. In addition, anakinra suppressed the expression of inflammatory biomarkers, such as IL-6, MMP-9, and MCP-1 in HUVEC, RAOSMC, and 3T3-L1 adipocytes, suggesting that anakinra treatment could be a useful strategy for blocking the inflammatory signals mediating the process of atherosclerosis and systematic inflammation. Anakinra produced a significant dose-dependent decrease in MMP-9 mRNA expression, and also decreased the migration of RAOSMCs, which is a model of vascular remodeling (Figure 8).

Anakinra was the first drug targeting IL-1 to be approved as a therapeutic agent for rheumatoid arthritis, and has now been recognized as having excellent long-term safety for treatment of chronic diseases such as cryopyrin-associated periodic syndromes, which are related to pathogenic variants of IL-1s [19,20]. Moreover, anakinra inhibited the expression of ICAM-1 and E-selectin in monocytes and improved endothelial dysfunction by reducing endoplasmic reticulum stress and the infiltration of inflammatory cells. It has also been reported to decrease the ischemia-induced neovascularization in diabetic rodents [21,22,23]. As the association between IL-1β and atherosclerosis has been highlighted, the benefits of targeting IL-1β has been emphasized in the past decades [24,25]. There has been supportive evidence in large scale clinical trials, for example, the Canakinumab (a monoclonal IL-1β antibody) Anti-inflammatory Thrombosis Outcome Study (CANTOS), found that it significantly lowered the recurrence rate in patients post-myocardial infarction at residual inflammatory risk of CVD as indicated by C-reactive protein (CRP) levels >2 mg/L despite the standard treatment strategy [26]. The analysis of the CANTOS trial showed that “responders” who were able to successfully reduce CVD events and mortality were characterized by the achievement of a reduction in CRP < 2 mg/L through IL-1β blockade. However, a recent experiment on the neutralization of two IL-1 isoforms showed different roles of IL-1α and IL-1β according to the stage of atherosclerosis in mice; IL-1α blockade affected the vascular remodeling during early atherosclerosis, but IL-1β blockade modulated inflammatory processes and reduced the extent of atheromatous plaque [27]. Meanwhile, IL-1β antibody treatment resulted in a significant decrease in smooth muscle cells and collagen substances and an increase in macrophages in the fibrous cap, leading to plaque instability in ApoE^–/–^ mice between 18 and 26 weeks of age on an atherogenic diet [28]. This study suggested that IL-1β per se might have multiple benefits in the late-stage murine atherosclerosis. Taken together, the effects of isoforms of IL-1 on the development and progression of atherosclerosis might be different, and the possibility of distinct effects depending on the stage of atherosclerosis should be kept in mind. As anakinra is a receptor blocker for IL-1, an extremely potent inflammatory cytokine, our results should be interpreted with caution in light of its blocking both IL-1 isoforms (IL-1α and IL-1β) signals, which might be similar yet distinct molecular pathways. In particular, it might be difficult to directly consider our results in line with the secondary prevention effect of blocking the IL-1 pathway on major adverse cardiovascular events after myocardial infarction in the CANTOS trial.

The ApoE^–/–^ mouse is a well-established animal model that has been applied extensively to research on the mechanism of atherosclerosis development [29]. ApoE^–/–^ mice have increased very-low density lipoprotein and increased LDL-C, an animal model that may reflect the poorly controlled residual risk of CVD in humans even during statin treatment. A previous study suggested that IL-1 blockade suppressed fatty-streak formation without interfering with the lipid metabolism of ApoE^–/–^ mice [30]. In addition, in the Ldlr^–/–^ (low-density lipoprotein receptor-deficient) mice, another representative atherosclerosis mouse model, anakinra stabilized atherosclerotic plaque remodeling making it less likely to rupture so that prevent ischemic events [31]. Interestingly, the present study showed that anakinra significantly reduced the level of serum TG in ApoE^–/–^ mice. In several IL-1 blockade studies in humans, improvements in lipid profiles including TG have been rarely reported, and have even been shown to increase TG or cholesterol levels [32,33]. As a decrease in serum TG was observed only at a relatively high dose of anakinra in this study, it is unclear whether improvement in atherosclerotic plaque was directly related to improvement in serum TG levels. However, further studies are needed, at least on the potential for improving lipid profiles along with the anti-inflammatory processes of IL-1 blockade.

The present study also showed that anakinra treatment reduced the burden of lipid-laden atherosclerotic plaques in ApoE^–/–^ mice by 30% compared with the control group. Similar results have been obtained using gevokizumab, a monoclonal antibody against IL-1β, which reduced plaque area in the aortic arch of ApoE^–/–^ mice by about 30% [24]. Moreover, anakinra induced a dose-dependent decrease in the number of CD68+ cells, widely used as a marker of the macrophage lineage, in fat tissue. Because inflamed fat tissue produces “bad” cytokines that promote systemic chronic inflammation, anakinra could provide a means by which to reduce both atherosclerotic plaques and inflammation in fat tissue [34,35,36,37].

The dysfunction and death of endothelial cells are known for their role in the initiation of atherosclerosis and recently, the NLRP3-mediated pathway has been identified as the main mechanism of pyroptosis in endothelial cells for atherosclerosis at early stages [38,39]. On the other hand, pyroptosis by NLRP3 inflammasome causes macrophage and SMC migration and induces foam-cell formation in the advanced stage of atherosclerosis, leading to necrotic core formation [40]. Therefore, based on the main action of NLRP3 inflammasome in the stage of atherosclerosis, it could be suggested that the anakinra dose and the effect on each cell line are different as presented in this study.

The present study found that the expression of IL-6 mRNA was lower in anakinra-treated HUVECs and RAOSMCs compared with control untreated cells. IL-6 is well recognized as a significant cytokine among several CVD-relevant inflammatory markers and is often known to be under the control of IL-1 in vitro and in vivo [41,42,43]. In this study, we demonstrated the effective inhibition of IL-6 through IL-1 blockade. In 3T3-L1 adipocytes, anakinra treatment resulted in significantly lower MCP-1 mRNA expression compared with the control group. MCP-1 is mainly produced by macrophages and endothelial cells, regulating the migration and infiltration of many inflammatory cells [44]. Since the gene expression of NLRP3 inflammasome was significantly reduced by anakinra treatment, it can be suggested that MCP-1 gene expression was also suppressed by a reduction in the inflammatory cascade [45]. In RAOSMCs, anakinra induced a significant dose-dependent decrease in MMP-9 expression, and also decreased RAOSMC migration, which reduces wound healing and the progress of vascular restenosis. This study also demonstrated that anakinra could inhibit the NF-κB and MAPK pathways to reduce atherosclerosis-prone inflammatory responses. A previous study using mice and rats with induced acute MI reported a 13% reduction in the infarct size in animals treated with 100 mg/kg anakinra, which suggested that IL-1 could play an important role in post-injury cardiac remodeling [13,23]. Taken together, these results suggest that anakinra inhibits the functions of IL-1, thereby reducing proinflammatory cytokine release and inflammatory cell recruitment, decreasing plaque size, and stabilizing the lipid-laden plaques, therefore, anakinra inhibits almost all stages of atherosclerosis.

This study has several limitations. First, the protocol specified that anakinra was administered at all stages, starting during a normal chow diet for the initial 4 weeks and continuing during an atherogenic diet for the last 12 weeks. This differs from other in vivo studies that used statin treatment only during an atherogenic diet. However, a previous study treating ApoE^–/–^ mice with IL-1 receptor antagonist showed that the drug affected the earlier stages of plaque formation [46]. Second, we wanted to clarify the effect of anakinra independent of the well-known effects of statin treatment in ApoE^–/–^ mice; thus, the combined effect of anakinra and statin could not be assessed. It is expected that anti-inflammatory mechanism-based treatment, anakinra, could additionally effect the inhibition of the initiation and progression of atherosclerosis in a variety of ways that are not possible with only some established lipid-lowering agents. Therefore, it would be helpful to conduct further studies in this animal model to investigate the anti-atherosclerosis effects of different doses and durations of combination treatment with anakinra and standard lipid-lowering agents. Third, anakinra showed a significant decrease in IL-1β mRNA expression in HUVEC, but did not show the clear effect on the expressions of ICAM-1 or MCP-1. Whether these were some characteristics of endothelial cells per se or the result of methodological issues such as anakinra dosage or time for treatment has not been confirmed in this study, but it is possible that other factors related to the subsequent process of endothelial cells intervened. Based on several previous studies showing increased gene expressions of cell adhesion molecules or monocyte-attracting chemokines, a number of factors such as the stimulus, growth factors, and collagens are thought to be involved in the modulation of endothelial cells [22,47]

## 4. Materials and Methods

### 4.1. Animals, Diet, and Treatment Protocols

All experimental protocols were approved by the Seoul National University Bundang Hospital Institutional Animal Care and Use Committee (BA1511-188/070-01). Animal experiments were performed in compliance with the guidelines from Directive 2010/63/EU of the European Parliament on the protection of animals used for scientific purposes or the NIH Guide for the Care and Use of Laboratory Animals.

Eight-week-old male ApoE^–/–^ mice of a C57BL/6 background (Jackson Laboratory, Bar Harbor, ME, USA) were fed a standard chow diet, pretreated with anakinra for 4 weeks, then fed an atherogenic diet containing 35 kcal% fat, 1.25% cholesterol, and 0.5% sodium cholic acid (D12236; Research Diets Inc., New Brunswick, NJ, USA) for 12 weeks with continued anakinra treatment. During the intervention period, the ApoE^–/–^ mice were divided into the following four treatment groups, where each drug was administered daily by intraperitoneal injection to assess the potential dose-dependent anti-atherosclerosis effects of anakinra: (1) control (*n* = 10, normal saline, 154 mmol/L NaCl), (2) anakinra 10 mg/kg (*n* = 10), (3) anakinra 25 mg/kg (*n* = 10), and (4) anakinra 50 mg/kg (*n* = 10). The number of mice per group was selected in accordance with a previous atherogenic experimental study [48]. A random sequence was created using Excel 2013 (Microsoft, Redmond, WA, USA). Mice were maintained in a controlled climate room with a light-dark cycle (12:12), and body weight and food intake were monitored once a week. At the time of euthanasia, mice were anesthetized by zoletil (30 mg/kg, i.p.) with xylazine (10 mg/kg, i.p.), and blood was collected by cardiac puncture after overnight fasting. Serum samples were used for triglyceride and cholesterol analyses. Triglyceride and cholesterol levels were measured by the Beckman Coulter AU480 automatic biochemistry analysis system (Tokyo, Japan). Aorta, liver, visceral fat, and muscle tissues were harvested for further histopathological analysis.

The aortic root was dissected longitudinally for the *en face* method and stained with oil-red O to measure the aortic atherosclerotic lesions. The entire aorta was removed and placed in 4% formaldehyde. The aorta was dissected using mini-Vanna scissors and forceps from the heart to the iliac bifurcation, pinned on a black wax dissection pan. To quantify the plaque area, the aortic arches were stained with Oil red O and hematoxylin. Section images were analyzed using an Olympus BX51 imaging system (Olympus, Tokyo, Japan) and quantified with Image-Pro Plus 6.0 software (MediaCybernetics, Bethesda, MD, USA). The area of atherosclerotic plaque was expressed as a percentage of the entire area of the aorta. The protocol for staining plaque fibrosis was described previously [49]. Further stains were performed in plaque areas using Masson’s trichrome, Sirus red, and α-SMA to evaluate fibrous caps, collagens, and differentiation of SMC, respectively. For the quantification of collagen contents, all sections were scanned with a photomicroscope (Axioskop 40, Carl Zeiss, Germany) and image files were analyzed using free, open-access program (QuPath software v0.3.0). The internal elastic membrane of tunica intima including atheroma plaques were analyzed using a positive pixel count for α-SMA.

### 4.2. Immunofluorescent Staining of CD68 and H&E Staining in the Adipose Tissue

The number of M1 macrophages in the fat tissue was evaluated as the number of CD68+ cells per 1 mm2, using an anti-mouse CD68 antibody (1:200 dilution, Abcam, Cambridge, MA, USA). H and E staining was also performed in the adipose tissue and the formation of crown-like structures were observed.

### 4.3. THP-1-Conditioned Media and Induction of NLRP3 Inflammasome Expression in HUVECs, RAOSMCs, and 3T3-L1 Cells

The human monocytic cell line, THP-1 (ATCC, Manassas, VA, USA; 5.5 × 106 cells/well) was maintained in RPMI 1640 (Gibco, Thermo Fisher Scientific, Waltham, MA, USA) supplemented with 10% fetal bovine serum (Gibco, Thermo Fisher Scientific, Waltham, MA, USA), at 37 °C and 5% CO_2_. To induce THP-1 cells to differentiate to macrophages, they were treated overnight with 100 nM phorbol-12-myristate-13-acetate (PMA; Sigma, Catalog #: P1585, Buchs, Switzerland), which was then replaced with fresh growth media and cultures were incubated for a further 24 h.

To examine the effect of anakinra on NLRP3 inflammasome activity in atherosclerosis, HUVECs (Lonza, San Diego, CA, USA), RAOSMCs (Bio-bud, Seoul, Republic of Korea), and 3T3-L1 cells (ATCC, Manassas, VA, USA) were used. Differentiated PMA-treated THP-1 cells were incubated for 6 h with 1 μg/mL (Sigma, Catalog #: L2880, Buchs, Switzerland) and 100 ng/mL TNF-α (ProSpec, Catalog #: Cyt-223-b, Ness-Ziona, Israel). Cells were removed (3000 rpm, 5 min, 0.22 μm filter) and supernatants (conditioned medium) were harvested. Then, the conditioned medium was added to pre-plated HUVECs, RAOSMCs, and 3T3-L1 in the presence or absence of anakinra.

### 4.4. Reverse Transcription–Quantitative Polymerase Chain Reaction (RT–qPCR)

The relative levels of mRNA transcripts for NLRP3, IL-1β, IL-6, MCP-1, ICAM-1, and MMP-9 were assessed with RT–qPCR using the β-actin gene as a reference. The sequences of the primers used are provided in online appendices (Supplementary Table S1).

### 4.5. Western Blot Analysis

Proteins were extracted from cells, and lysates containing appropriate amounts of protein were resolved on 10% SDS-polyacrylamide gels and transferred to polyvinylidene difluoride membranes. Nonspecific binding was blocked in 5% bovine serum albumin for 2 h at room temperature. Membranes were incubated overnight at 4 °C with primary antibodies (Supplementary Table S2). Next, the membranes were washed and then incubated for 1 h at room temperature with horseradish peroxide-conjugated anti-rabbit or anti-mouse secondary antibodies (Santa Cruz Biotechnology, Dallas, TX, USA). The densitometric quantification of the protein bands was determined with the ImageJ software version 1.29x (National Institutes of Health, Bethesda, MD, USA).

### 4.6. RAOSMC Migration Assays

RAOSMC migration capacity was assessed with two-dimensional wound healing assays. For the wound-healing assay, cells were seeded at a density of 2 x 10^5^ cells/well in 12-well plates and starved with serum-free DMEM media for 24 h before experiments. Linear wounds were made by scratching with a 1000 mL pipette tip. RAOSMCs were allowed to migrate for 24 h in the presence or absence of PDGF (10 ng/mL) and anakinra (1000 ng/mL) at 37 °C; then images of the migrated RAOSMCs were acquired using an inverted microscope (Olympus, Tokyo, Japan).

### 4.7. Statistical Analysis

All data are expressed as mean ± standard error of the mean. Statistical significance was determined using the analysis of variance (ANOVA) with Tukey’s post hoc analysis for multiple group comparison. Values of two-sided *p* < 0.05 were considered significant. Statistical analyses were performed using SPSS Statistics for Windows (version 24.0; IBM Corp., Armonk, NY, USA). Illustration was created using the online software tool (BioRender, Toronto, ON, Canada).

## 5. Conclusions

We demonstrated that the IL-1 blocker anakinra produced a 30% reduction in atherosclerotic plaque area, TG levels and macrophage infiltration in adipose tissue in ApoE^–/–^ mice on atherogenic diet, and confirmed its anti-inflammatory effects in experiments using RAOSMC, HUVEC, and 3T3-L1 adipocytes. Atherosclerotic CVDs have a complicated pathogenesis, so identifying the best way to ameliorate the residual risk after standard therapy for preventing CVD events remains a challenge. Our results are mediated by blocking chronic inflammation in atherosclerosis via the IL-1 cascade, and could support the notion that anti-inflammatory, novel targeted cytokine-based therapies together with lipid-lowering agents could be considered to overcome the residual risk of major cardiovascular events.

## Data Availability

The datasets used and/or analyzed during the current study are available from the corresponding author upon reasonable request.

## Supplementary Materials

The following supporting information can be downloaded at: https://www.mdpi.com/article/10.3390/ijms23094906/s1.

## Abbreviations and Acronyms

ANOVA	Analysis of variance
ApoE^–/–^	Apolipoprotein E knockout
CRP	C-reactive protein
CVD	Cardiovascular disease
ERK	Extracellular-regulated kinase
H&E	Hematoxylin and eosin
HUVEC	Human umbilical vein endothelial cell
ICAM	Intracellular adhesion molecule
IL	Interleukin
LDL-C	Low-density lipoprotein cholesterol
LPS	Lipopolysaccharide
MAPK	Mitogen-activated protein kinase
MCP-1	Monocyte chemoattractant protein-1
MI	Myocardial infarction
MMP-9	Matrix metalloproteinase-9
NF-κB	Nuclear factor kappa-light-chain-enhancer of activated B cell
NLRP3	NACHT, LRR, and PYD domain-containing protein 3
PDGF	Platelet-derived growth factor
P-JNK	Phosphorylated c-Jun N-terminal kinase
RAOSMC	Rat aortic smooth muscle cell
SMA	Smooth muscle actin
TG	Triglycerides
TNF-α	Tumor necrosis factor-α.
