# Supplementary material for: The Anti-Atherosclerosis Effect of Anakinra, a Recombinant Human Interleukin-1 Receptor Antagonist, in Apolipoprotein E Knockout Mice
Source: Int J Mol Sci. 2022 Apr 28;23(9):4906. doi: 10.3390/ijms23094906 (PMC9104865; doi:10.3390/ijms23094906)

Supplementary Materials

The anti-atherosclerosis effect of anakinra, a recombinant human interleukin-1 receptor antagonist, in apolipoprotein E knockout mice

Eu Jeong Ku[1,2,†], Bo-Rahm Kim[3,†], Jee-In Lee[3], Yun Kyung Lee[3], Tae Jung Oh[3,4], Hak C. Jang[3,4] and Sung Hee Choi[3,4*]

[1] Department of Internal Medicine, Chungbuk National University Hospital, Cheongju 28644, Korea; eujeong.ku@gmail.com
[2] Department of Internal Medicine, Chungbuk National University College of Medicine, Cheongju 28644, Korea; eujeong.ku@gmail.com
[3] Department of Internal Medicine, Seoul National University Bundang Hospital, Seongnam 13620, Korea; cjlovem@naver.com (B.-R.K.); dlwldls715@naver.com (J.-I.L.); leeykyung@gmail.com (Y.K.L.); ohtjmd@gmail.com (T.J.O.); janghak@snu.ac.kr (H.C.J.); shchoimd@gmail.com (S.H.C.)
[4] Department of Internal Medicine, Seoul National University College of Medicine, Seoul 03080, Korea.; ohtjmd@gmail.com (T.J.O.); janghak@snu.ac.kr (H.C.J.); shchoimd@gmail.com (S.H.C.)
* Correspondence: shchoimd@gmail.com; Tel.: 82-31-787-7033
† These authors contributed equally to this work as co-first authors.

Contents

- **Table S1. Primers used in the study**
- **Table S2. Antibodies used for western blot assays**
- **Figure S1. Western blot of important markers in (a) HUVEC, (b) RAOSMC, and (c) 3T3-L1 adipocytes.**
- **Figure S2. Expression of phosphorylated c-Jun N-terminal kinase (p-JNK), p-p38, and p-ERK in the (a) liver and (b) visceral fat tissue.**

Table S1. Primers used in the study

Gene	Type	Forward sequence	Reverse sequence
IL-1β	Human	ATGATGGCTTATTACAGTGGCAA	GTCGGAGATTCGTAGCTGGA
IL-1β	Murin	GAAATGCCACCTTTTGACAGTG	TGGATGCTCTCATCAGGACAG
IL-1β	Rat	CTGTGACTCGTGGGATGATG	GGGATT TTG TCG TTG CTT GT
NLRP3	Human	GATCTTCGCTGCGATCAACAG	CGTGCATTATCTGAACCCCAC
NLRP3	Murin	ATTACCCGCCCGAGAAAGG	CATGAGTGTGGCTAGATCCAAG
NLRP3	Rat	GCTGCTCAGCTCTGACCTCT	AGGTGAGGCTGCAGTTGTCT
IL-6	Human	TACCCCCAGGAGAAGATTCC	TTTTCTGCCAGTGCCTCTTT
IL-6	Murin	CTGCAAGAGACTTCCATCCAG	AGTGGTATAGACAGGTCTGTTGG
MCP-1	Human	TCT GTG CCT GCT GCT CAT AG	CAG ATC TCC TTG GCC ACA AT
MCP-1	Murin		
ICAM-1	Human	ATG CCC AGA CAT CTG TGT CC	GGG GTC TCT ATG CCC AAC AA
MMP-9	Rat	CAAACCCTGCGTATTTCCAT	AGTTGCCCCCAGTTACAGTG
β-actin	Rat	AGC CAT GTA CGT AGC CAT CC	CTC TCA GCT GTG GTG GTG AA

Table S2. Antibodies used for western blot assays

Antibody for proteins	Manufacturers	Catalog numbers
Phospho-p65	Cell Signaling Technology	3033
p65	Santa Cruz Biotechnology	sc-7151
Phospho-p38	Cell Signaling Technology	9211
p38	Cell Signaling Technology	9212
Phospho-ERK	Cell Signaling Technology	9101S
ERK	Cell Signaling Technology	9102S
Phospho-JNK	Cell Signaling Technology	9251S
JNK	Cell Signaling Technology	9252S
IL-1β	Abcam	ab254360
TNF-α	Invitrogen	PA5-19810
IL-6	Abcam	ab259341
MMP-9	Abcam	38898
ICAM-1	Santa Cruz Biotechnology	sc-107
γ-tubulin	Sigma-Aldrich	T6199
β-actin	Sigma-Aldrich	A5441

Figure S1. Western blot of important markers in (a) HUVEC, (b) RAOSMC, and (c) 3T3-L1 adipocytes.

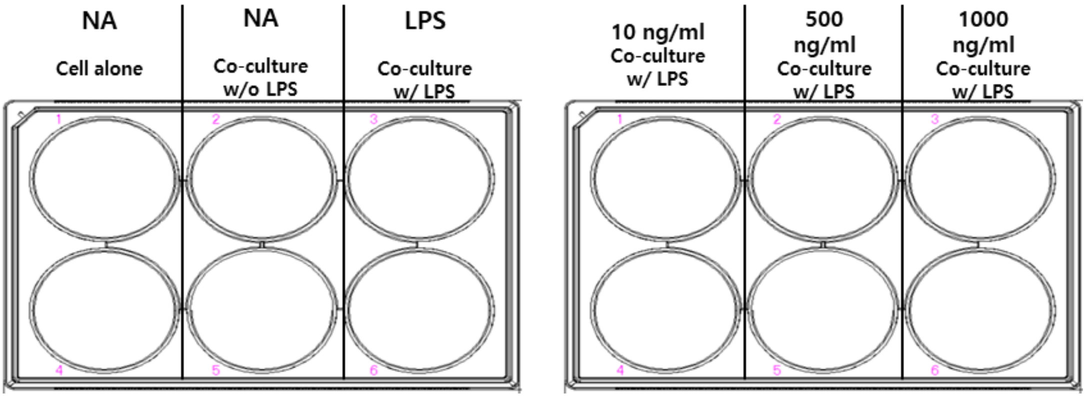

a. HUVECs

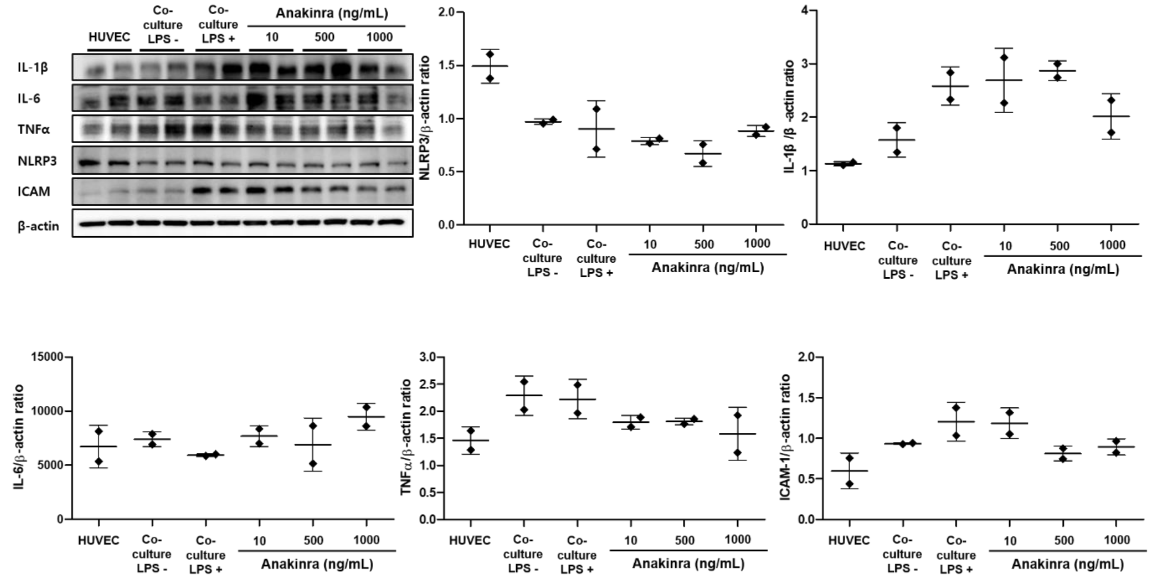

b. RAOSMCs

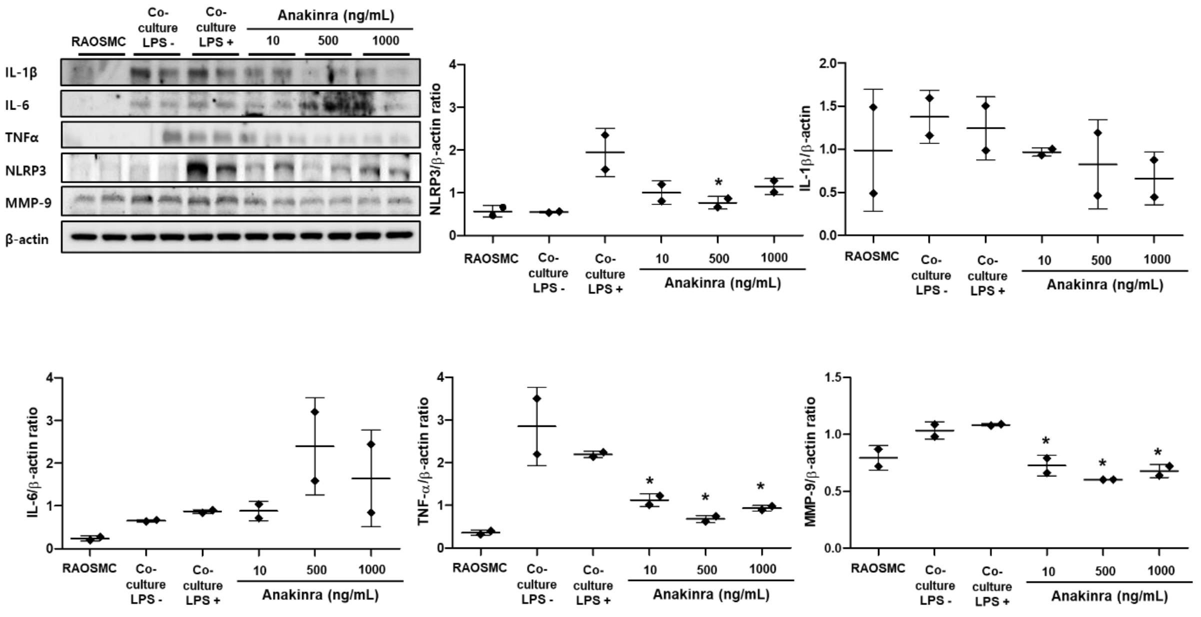

c. 3T3-L1 adipocytes

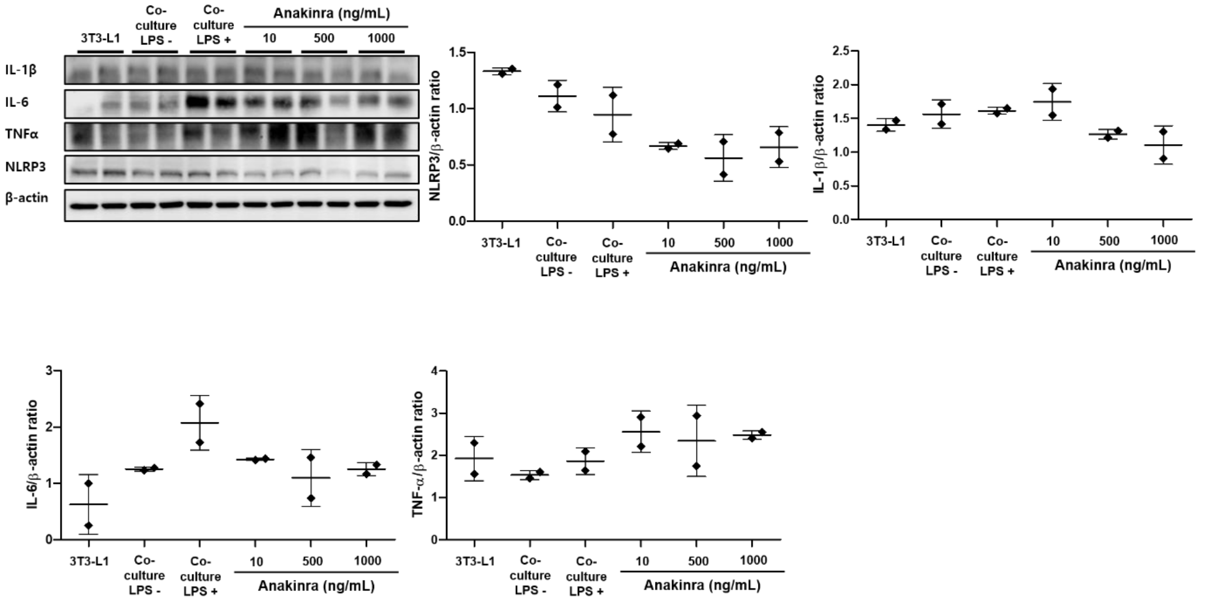

Figure S2. Expression of phosphorylated c-Jun N-terminal kinase (p-JNK), p-p38, and p-ERK in the (a) liver and (b) visceral fat tissue.

a. Liver

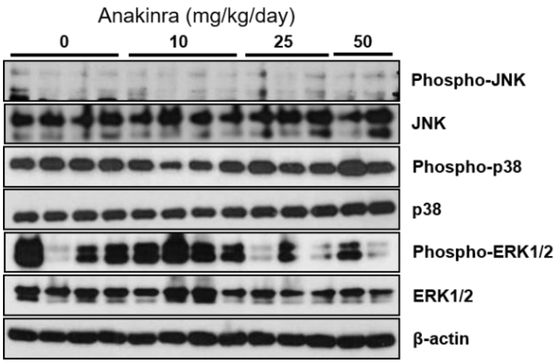

b. Visceral fat tissue

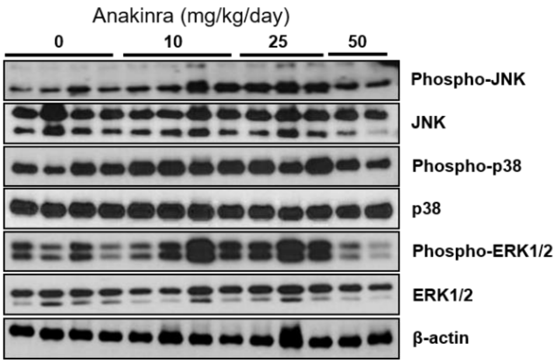

Supplement: Supplementary file 1 [file ijms-23-04906-s001.zip › ijms-1700378-supplementary.pdf]
